# Effect of the COVID-19 Pandemic on Gambling Behavior in Mainland Chinese Gamblers in Macau: Cross-Sectional Survey Study

**DOI:** 10.2196/45700

**Published:** 2024-04-22

**Authors:** Jinquan Zhou, Hong-Wai Ho, ChiBiu Chan

**Affiliations:** 1 Macao Polytechnic University Macao China; 2 Centre for Gaming and Tourism Studies Macao Polytechnic University Macao China; 3 Faculty of Business Macao Polytechnic University Macao China

**Keywords:** Chinese gamblers, gambling behavior, online gambling, COVID-19, Macau

## Abstract

**Background:**

This study examined the effects of the COVID-19 pandemic on the gambling behavior of individuals who were already actively engaged in such pursuits. We aimed to uncover the intricate consequences of the pandemic on this specific demographic, emphasizing the importance of understanding the complex connection between public health concerns such as the COVID-19 pandemic and gambling behavior from a public health perspective. In addition to identifying immediate impacts, this study holds significance in assessing potential long-term public health implications for the broader gambling industry.

**Objective:**

This study investigated how the COVID-19 pandemic has affected the gambling behavior of Mainland Chinese tourists in Macau from a public health perspective. We aimed to understand the changing patterns of gambling habits within this specific demographic by comparing their behavior before and during the pandemic, with a particular emphasis on the evolving dynamics of gambling and their public health consequences. This study provides a detailed exploration of the impact and implications of global health emergencies on this particular demographic’s gambling behaviors and preferences.

**Methods:**

This study used a robust cross-sectional analysis involving a sample of 334 Mainland Chinese gamblers with prior experiences in casinos in Macau. The sample deliberately encompassed individuals involved in gambling before and during the COVID-19 pandemic. Data were collected through carefully designed questionnaires to gather information on gambling habits, preferences, and observed behavioral changes in the sample.

**Results:**

This study unveiled a notable shift in Mainland Chinese gamblers’ behavior during the COVID-19 pandemic. A considerable number of participants opted for web-based platforms over traditional land-based casinos, resulting in reduced budgets, less time spent on gambling, and decreased participation in social gambling. Remarkably, there was a notable surge in online gambling, indicating a noteworthy adaptability of gamblers to changing circumstances. These findings emphasize the dynamic nature of gambling habits during global public health emergencies, revealing the resilient and evolving preferences of Mainland Chinese gamblers in response to the challenges posed by the pandemic.

**Conclusions:**

This study highlights the negative impact of the COVID-19 pandemic on casino gambling, notably evident in a significant decline in Mainland Chinese tourists visiting Macau for gambling. There is a noticeable shift from traditional gambling to web-based alternatives, with individuals seeking options within the pandemic constraints. Furthermore, the findings point out an increase in gambling among the younger generation and behavioral changes in individuals with mood disorders. The findings of this study emphasize the critical need for proactive measures to address evolving gambling preferences and associated risks during public health crises; furthermore, these findings underscore the importance of adaptive strategies within the gambling industry, as well as the necessity for effective public health interventions and regulatory frameworks to respond to unprecedented challenges with efficacy and precision.

## Introduction

### Background

Gambling addiction is widely acknowledged as a significant public health concern [1,2]. Some common types of gambling-related harm include financial difficulties, mental health problems, relationship disruption, emotional distress, cultural harm, and also involvement in criminal activities [3]. In Macau, gambling plays a vital role in driving economic growth, tourism, and job creation. Since 2006, Macau has risen to prominence as the world’s largest gambling market, surpassing Las Vegas [4,5], with Mainland Chinese tourists constituting a substantial 93% of the city's gambling clientele [6]. While this success has undoubtedly contributed significantly to tourism and economic development, it has also brought forth a concerning issue—the rise of gambling addiction among the influx of Chinese tourists. Despite their pivotal role in Macau’s prosperity, the substantial number of Chinese tourists engaging in gambling activities raises pressing concerns regarding the prevalence of problem gambling within this demographic.

The COVID-19 pandemic, caused by SARS-CoV-2, has been a global health crisis since late 2019 and continues to affect the world today [7]. Similar to many other regions, Macau has not been immune to the rapid spread of the virus. In response, the region implemented stringent measures to control the transmission, including a mandatory 14-day quarantine and a required nucleic acid test for entrants. This pandemic has evolved into a worldwide public health crisis, with profound economic repercussions on a global scale [8]. Macau’s gambling industry, in particular, faced significant challenges, experiencing a substantial decrease in gambling revenue [9]. This decline underscores the impact of the pandemic on gamblers’ behavior and the challenges faced by the sector due to public health measures. The highly contagious nature of the coronavirus prompted a crisis in environmental health for tourists and gamblers [10]. In response, public health authorities prioritized anti–COVID-19 measures, ranging from recommendations to enforceable mandates. In Macau, the local government adopted a stringent approach, implementing a zero-tolerance policy with mandatory quarantine and nucleic acid testing at entry and exit points. Furthermore, casino operators adopted measures to limit the concentration of gamblers and reduce the risk of virus transmission. Aligned with the Chinese government, the authorities in Macau implemented a health code system for public health and safety. The COVID-19 pandemic significantly impacted the casinos in Macau, leading to lasting negative effects on the gambling and tourism industries. Some casinos were forced to close due to the challenges posed by the pandemic [11].

The growing global prevalence of gambling and its potential impact on public health prompted us to analyze the evolving patterns of gambling. Chinese gamblers constitute the primary course of patronage for casinos across Asia [12]. While gambling is prohibited in Mainland China, Macau stands out as a legal gaming destination due to its Special Administrative Region (SAR) status under the “one country, two systems” principle. This study holds particular relevance in understanding the behavior of Chinese gamblers within regional and global public health environments. The COVID-19 pandemic has significantly influenced gambler behavior, including factors such as travel restrictions [13], risk assessment [14], alternative attractions, the impact of destination marketing strategies [15], and the rise of online gambling [16]. Macau’s distinctive position as a major gambling hub, heavily dependent on tourism, presents an intriguing case to investigate the public health implications of COVID-19 and gambling addiction. However, there is a notable research gap in understanding the changes in gambling behavior among Mainland Chinese gamblers in Macau during the pandemic. Examining these shifts is crucial to addressing the existing knowledge gap in this context and gaining a comprehensive understanding of the effects of the pandemic on gambling patterns and preferences. This knowledge will enable the implementation of evidence-based public health policies and practices that prioritize the well-being and safety of both local residents and tourists in the postpandemic era.

We aimed to investigate the gambling behaviors of Chinese gamblers during the COVID-19 pandemic, specifically by comparing their past and current behaviors through a cross-sectional analysis. Furthermore, this study explores the factors that influence these behaviors within the context of public health measures. The findings offer valuable insights and recommendations for industry executives and policy makers, facilitating a comprehensive understanding of the shifts in gambling patterns during public health crises. Considering the importance of public health, these insights could also serve as a crucial resource for guiding strategic adjustments in the gambling industry.

### The Literature

#### COVID-19 Impact on Tourism: Historical Context and Consequences in Macau

Gambling is a recognized public health issue with wide-ranging implications. The recent COVID-19 pandemic has brought forth significant public health implications for the gambling industry worldwide [16]. In response to the pandemic, the Macau SAR government implemented various proactive measures to safeguard public health, including specific guidelines and regulations for casinos [17]. Following the confirmation of the first COVID-19 case on January 22, 2020, the local government temporarily suspended casino and entertainment businesses for half a month starting February 4, 2020. Furthermore, nonessential services were postponed, with entry bans for nonresidents implemented from March 18, 2020, except for individuals from Mainland China, Hong Kong, Taiwan, and nonresident workers. Effective from May 11, 2020, entry requirements included a negative COVID-19 test within 7 days or a Macau Guangdong Health Code [18]. Macau gradually resumed normal casino operations in August 2020, but foreign tourists were required to undergo nucleic acid testing within 7 days before arrival. Preventive measures within casinos included mask-wearing mandates, exclusion of individuals with fever or acute cough, health declarations, and health codes. Entry was denied to those with influenza-like symptoms, and visitors’ health conditions were closely monitored. Inside the casinos, efforts were made to prevent close contact, avoid overcrowding, suspend promotional activities, and limit the number of individuals to ≤50% of the venue’s capacity. A minimum distance of 1 m was maintained between gamblers.

The ongoing pandemic and the subsequent prevention strategies have contributed to the resurgence of travelers visiting Macau for gambling and leisure purposes. The rapid transmission of COVID-19 has led to a decline in consumer economic activity, directly impacting the gambling and tourism industry. The COVID-19 pandemic has had a devastating effect on the global tourism sector, as strict measures have been taken to contain the spread of SARS-CoV-2. Consequently, there has been a substantial decline in global tourist arrivals [19]. For instance, in Macau, total visitor spending (excluding gambling) in the first quarter of 2020 experienced a substantial year-on-year decline of 70.4%, reaching MOP 5.01 billion (MOP $1=US $0.12). Per capita expenditure also decreased by 4.8%, with each visitor spending approximately MOP 1555 during that period [20].

On the basis of the aforementioned information, it is evident that COVID-19 has had a significant impact on consumer behavior and public health, particularly in relation to the gambling industry and the broader tourism sector. The pandemic has resulted in negative effects on gambling spend and public health, such as a decrease in gambling revenue, changes in the consumption patterns of tourists, and a decline in the overall tourism industry [21]. Moreover, the public health implications of COVID-19 have not only affected the emotional and psychological well-being of gamblers but also led to changes in their behavioral patterns due to safety concerns [22]. The altered behavior and psychological needs resulting from the COVID-19 pandemic are likely to influence the perception of safety among tourists and subsequently affect their willingness to gamble and their gambling behavior from a public health perspective.

#### Gambling Behavior

Gambling is part of a gambler’s leisure activities and social relations [23]. Given the unique characteristics of gambling compared to other consumption behaviors, the gambling process carries the possibility of influencing income. Rational individuals who engage in gambling derive enjoyment from the overall journey of the activity, regardless of whether they win or lose money during the process. Gambling as a consumption item exhibits a distinct attribute wherein the potential for income fluctuation during consumption is observed [24]. Gambling is recognized as an activity through which individuals have the opportunity to pursue potential financial gains or explore avenues for earning income [25]. Furthermore, it is essential to consider gambling behavior as a continuous variable. The involvement of individuals in gambling displays inherent flexibility, indicating the potential for modifications influenced by various factors. Within this continuum, it is noteworthy that gamblers often demonstrate a propensity for frequent gambling [1] and engage in a wider variety of games [26,27].

Gambling behavior is associated with gambling experience, frequency, and morbidity [28-30]. More frequent gambling, participation in more types of gambling, and the cost of gambling are associated with pathological gambling behavior [26,27]. The South Oaks Gambling Screen, developed by Lesieur and Blume [31], was designed to measure and assess pathological gambling behavior, particularly focusing on gambling frequency. Raylu and Oei [32] developed the Gambling Urge Scale to measure pathological gambling behavior with gambling adventures. Hasking and Oei [33] tested the pressure measurement of pathological gambling behavior using COPE questionnaire.

Moreover, gambling behavior is influenced by several factors, including psychological activity and the psychological characteristics of participation in gambling [34]. In particular, the theory of adaptation is used as a supplement to the exposure hypothesis to study the impact of the environment on gambling behavior. Social adaptation will lead gamblers to gradually realize that their chances of winning money are negative in the long run. In response, gamblers adjust the amount of money they bet and even their gambling behavior (such as abandoning gambling). People’s prolonged lack of exposure to gambling facilities can lead to a gradual reduction in interest in gambling games or a modification of their behavior [35].

In addition, gambling is a limited rational economic behavior influenced by the environment and culture. Binde [36] noted that due to cultural and historical influences, gambling participation has not improved despite various restrictions. After analyzing the psychological characteristics of gambling participants, they found that gambling intention leads to attributes such as the concept of luck, the neglect of probability, the “illusion of success,” and “the illusion of control.” Participation will be influenced by personal psychological characteristics such as risk aversion, ambiguity aversion, risk tolerance, loss aversion, and optimism. However, the National Research Council found that approximately 98% of Americans had no clinical problems [37]. The association between gambling intention and the frequency of gambling, as well as problem gambling, has been consistently observed in research studies [38,39]. Despite the implementation of diverse restrictions, it is important to acknowledge that participation in gambling behavior has not witnessed significant improvement, which can be attributed to the influence of cultural and historical factors [40]. In the realm of gambling research, it is widely acknowledged that gambling behavior is intricately linked to both the experience and frequency of gambling [28,30]. These prior studies highlight the significance of individuals’ prior exposure to gambling activities as a key determinant influencing their subsequent gambling behavior. Consequently, researchers have introduced the variable of experience as an additional determinant to elucidate the intricate complexities underlying gambling behavior [41].

Chinese gamblers have unique gambling characteristics [42]. The *Diagnostic and Statistical Manual of Mental Disorders* (Fifth Edition), defines gambling disorder as a behavioral addiction characterized by “persistent and recurrent problematic gambling behavior leading to clinically significant distress or impairment” [43]. Despite most forms of gambling being banned in Mainland China (except for state-run lotteries), the prevalence of gambling addiction in Mainland China is estimated to be higher than that in many Western countries, affecting approximately 2.5% to 4% of the adult population [44]. The participation rate in various forms of gambling in China is relatively high, which may be influenced by the cultural acceptance of gambling [24]. This cultural acceptance of gambling as a form of entertainment and social activity has contributed to its widespread practice among Chinese individuals [45-47]. However, it is important to acknowledge that the Chinese government has been making considerable efforts to combat illegal cross-border and online gambling activities, aiming to mitigate potential negative consequences such as financial strain and addiction [48,49].

Familiarity and recognition of gambling are the factors that attract Chinese individuals to casinos [50]. In contrast to tourists from various regions [6], the Chinese community stands out for its strong emphasis on gambling, as it is widely regarded as a preferred form of entertainment [45]. Furthermore, the Gambling Among Members of Ethnic Communities in Sydney Project found that Chinese participants thought gambling was a routine social activity rather than a way to escape the problems of everyday life [51]. A multisite study on older Chinese-Canadian gambling patterns and associated predictors showed that higher education levels and higher life satisfaction reduced the likelihood of gambling [52]. Some studies have found that Chinese (especially men) individuals have higher rates of gambling and problem gambling than the general population [53]. Chinese people who participate in gambling spend more money per week than the general community, and the participation rate of casino game consoles in the Chinese community is lower than that in other communities [54]. Galletti [55] reported that Chinese gamblers often gamble differently than the average North American gambler. Chinese gamblers often adjust their bets drastically based on their perception of luck, essentially outperforming the average North American gambler regarding the frequency of participation in various forms of gambling and gambling bets [32].

The COVID-19 pandemic has had several notable effects on gambling behavior from a public health standpoint. First, previous studies have reported significant reductions in the number of individuals engaging in gambling activities due to the pandemic and public health interventions [22,56]. In addition, the implementation of travel restrictions aligned with public health recommendations has influenced gambling behavior by imposing limitations on the gamblers’ ability to travel to their preferred destinations [13]. Furthermore, individuals have been more cautious in assessing the risks associated with traveling abroad [14], leading to considerations of alternative attractions or tourism products that notably increased during the pandemic [16]. In addition to the aforementioned impacts, there has been a notable increase in online gambling, particularly among the younger generation [22]. It is also important to note that during the COVID-19 pandemic, online gambling was associated with an elevated risk of problem gambling [57].

## Methods

### Study Design

This study used a quantitative design to investigate the gambling behavior of Mainland Chinese gamblers in Macau during the COVID-19 pandemic. Given the challenges of distributing questionnaires directly to individuals amidst the pandemic, a web-based data collection method was used. The survey team consisted of members from the marketing department of Macau casinos who actively engaged with customers through their social instant SMS text messaging platform (WeChat groups, Tencent Holdings Limited). This approach allowed the study team to effectively gather data on the gambling activities of these customers in Macau both before and after the pandemic.

### Data Collection

Owing to the travel restrictions imposed during the COVID-19 pandemic, conducting on-site questionnaire distribution became challenging. Therefore, this study used web-based surveys through the social instant SMS text messaging platform “WeChat” to investigate gamblers who had visited Macau to gamble before the pandemic and whether they had visited Macau during the pandemic. The study used a carefully designed questionnaire using a closed-question format to gather data. To ensure a representative sample, a targeted sampling approach was adopted to reach out to individuals with relevant experiences and characteristics regarding the research topic. The survey was conducted from February 24 to March 2, 2021. A total of 350 questionnaires were distributed among the active groups on WeChat, a popular instant SMS text messaging and social media platform in Mainland China. Following a careful review of the responses received, 334 questionnaires were considered valid, representing a response rate of 95.4%.

### Measures

We used the following measurement tools to assess gambling behavior in this study. The South Oaks Gambling Screen, developed by Lesieur and Blume [31] in 1987, was used to measure pathological gambling behavior based on gambling frequency. The Gambling Urge Scale [32] was used to measure pathological gambling behavior characterized by excessive risk-taking [58]. The COPE (Pressure Measurement of Pathological Gambling Behavior Scale) was used to assess the pressure associated with pathological gambling behavior [33]. In addition, the gambling experience scale is used to gauge various aspects of gambling behavior [28,29,59]. The gambling behavior scale encompassed the following 3 dimensions: gambling amount (3 items: “I often choose to bet large amounts when I gamble”; “I can control the amount of money I bet”; and “I can bet on budget when I gamble”); gambling mood (3 items: “when I am depressed”; “I will increase the number of times I engage in gambling when I feel stressed and nervous”; and “I will prolong my gambling time, I feel self-blame for my gambling behavior and consequences”); and social gambling (3 items: “I can give up or delay critical social activities because I turn to gambling”; “If someone invites me to gamble together, I will not hesitate to get involved”; and “I will most likely participate in gambling-related activities if it is necessary for socializing”).

The basic sociodemographic variables included in this study were sex (female or male); marital status (married or unmarried); age (grouped into 3 categories: ≤25 years, 26-35 years, ≥36 years); monthly income (divided into 4 groups: monthly income of <RMB 10,000; RMB 10,001 to RMB 20,000; RMB 20,001 to RMB 30,000; and ≥RMB 30,001); education level (categorized as high school or below, junior college degree, and bachelor’s degree or above); and gambling before the age of 21 years (indicated as either yes or no).

In addition to the sociodemographic variables, the respondents were asked to provide information about their behavior during the COVID-19 pandemic. The questionnaire included the following items: participation in gambling activities during the pandemic (with options of yes or no); online gambling during the pandemic (with options of yes or no); prepandemic gambling frequency (once a week, once a month, once a day, or irregular); gambling budget for each prepandemic participation (<RMB 10,000, RMB 10,001 to RMB 20,000, RMB 20,001 to RMB 30,000, ≥RMB 30,001, or no budget); gambling budget for each gambling participation after the pandemic (<RMB 10,000, RMB 10,001 to RMB 20,000, RMB 20,001 to RMB 30,000, ≥RMB 30,001, or no budget); duration of stay at the casino (<6 hours, 6-12 hours, 1 day-2 days, 2-3 days, or >3 days); and habits (with options of smoking, drinking, not smoking, or not drinking).

### Analysis

The methodological approach of this study involved the use of an web-based survey design to collect data on gambling behavior from Mainland Chinese gamblers. Specifically, this study focused on examining both online gambling and on-site gambling activities. The survey was developed and distributed through WeChat groups and consisted of 3 sections. The first section encompassed statements related to on-site and online gambling and other gambling activities. This section included questions about the frequency of gambling in the past year and the gambling budget both before and during the pandemic. The second section used 9 scales to measure various aspects of gamblers’ behavior. Respondents were asked to rate their responses on a 5-point scale. The last section of the survey was dedicated to collecting demographic data from the respondents and providing information about their characteristics.

### Ethical Considerations

The methodology and survey questionnaire used in this study were approved by the institutional review board of the Center for Gaming and Tourism Studies, Macao Polytechnic University (ethics approval number: RP/CJT-02/2023/E01). All procedures complied with the 1964 Helsinki Declaration and its later amendments or comparable ethical standards. The key aspects of the research ethics of human subjects are confidentiality, privacy, and consent. We obtained informed consent from all participants after fully informing them about the study. Privacy and confidentiality of participants’ personal information were strictly maintained throughout the research process, with data anonymization techniques used during reporting to protect their identities. Throughout the study, attention was given to addressing any potential conflicts of interest and upholding the highest ethical standards to protect the rights of the participants. No compensation was provided for participation in any aspect of the study.

## Results

### Sample Characteristics

The descriptive data for the sample is presented in Table 1. Among the 334 respondents, 49.1% (n=164) reported participating in gambling activities before the age of 21 years, while 50.9% (n=170) indicated that they did not engage in such activities. Of the 334 respondents, 48.8% (n=163) indicated their involvement in casino gambling activities during the pandemic, while 51.2% (n=171) reported abstaining from such activities. Furthermore, 52.4% (175 /334) of the respondents reported participating in online gambling, whereas 47.6% (159 /334) individuals reported nonparticipation in online gambling. Of the respondents, 64.4% (215/334) of individuals identified as male. Regarding educational attainment, 24.9% (83/334) of the respondents indicated that their education level was high school or below, 41.6% (139/334) respondents reported that they were enrolled in college, and 33.5% (112/334) respondents held bachelor’s degrees or higher qualifications. A substantial majority of participants, 77.5% (259/334) stated that they were married.

In terms of age distribution, the largest proportion of respondents (192/334, 57.5%) were aged 26 to 35 years, followed by those aged 36 to 50 years (96/334, 28.7%). Regarding monthly income, most respondents (177/334, 53%) earned <RMB 10,000; in addition, 95 (28.4%) of the 334 respondents reported a monthly income between RMB 10,001 and RMB 20,000, while 44 (13.2%) fell within the income range of RMB 20,001 to RMB 30,000.

Before the pandemic, the gambling frequency of the 334 respondents varied as follows: 126 (37.7%) individuals engaged in gambling once a month, 81 (24.3%) individuals gambled once a week, 23 (6.9%) individuals gambled once a day, and 104 (31.3%) individuals gambled irregularly. As for the allocation of the gambling budget, 202 (60.5%) respondents allocated <RMB 10,000, 76 (22.8%) respondents allocated between RMB 10,001 and RMB 20,000, 45 (13.5%) respondents allocated between RMB 20,001 and RMB 30,000, and 11 (3.3%) respondents allocated ≥RMB 30,001.

During the pandemic, the gambling budget distribution among the 334 respondents was as follows: 156 (46.7%) respondents allocated <RMB 10,000, 79 (23.7%) respondents allocated between RMB 10,001 and RMB 20,000, 37 (11.1%) respondents allocated between RMB 20,001 and RMB 30,000, 18 (5.4%) respondents allocated ≥RMB 30,001, and 44 (13.2%) respondents did not have any specific budget.

**Table 1:** Profile of the sample (N=334).

Item and response		Frequency, n (%)	Cumulative (%)
**Gambling before the age of 21 years**			
	Yes	164 (49.1)	49.1
	No	170 (50.9)	100
**Gambling in casino**			
	Yes	163 (48.8)	48.8
	No	171 (51.2)	100
**Gambling online**			
	Yes	175 (52.4)	52.4
	No	159 (47.6)	100
**Gender**			
	Male individuals	215 (64.4)	64.4
	Female individuals	119 (35.6)	100
**Education**			
	High school and below	83 (24.9)	24.9
	Junior college	139 (41.6)	66.5
	Bachelor’s degree and above	112 (33.5)	100
**Habit**			
	Smoking	91 (27.2)	27.2
	Drinking	102 (30.5)	57.8
	None of the above	141 (42.2)	100
**Marital status**			
	Married	259 (77.5)	77.5
	Unmarried	75 (22.5)	100
**Gambling frequency (before pandemic)**			
	Once a week	81 (24.3)	24.3
	Once a month	126 (37.7)	62
	Once a day	23 (6.9)	68.9
	Irregular	104 (31.3)	100
**Age (years)**			
	≤25	46 (13.8)	13.8
	26 to 35	192 (57.5)	71.3
	≥36	96 (28.7)	100
**Monthly income^a^**			
	≤RMB 10,000	177 (53)	53
	RMB 10,001 to RMB 20,000	95 (28.4)	81.4
	RMB 20,001 to RMB 30,000	44 (13.2)	94.6
	≥RMB 30,001	18 (5.4)	100
**Gambling budget (before the pandemic)**			
	≤RMB 10,000	202 (60.5)	60.5
	RMB 10,001 to RMB 20,000	76 (22.8)	83.2
	RMB 20,001 to RMB 30,000	45 (13.5)	96.7
	≥RMB 30,001	11 (3.3)	100
**Gambling budget (during the pandemic)**			
	≤RMB 10,000	156 (46.7)	46.7
	RMB 10,001 to RMB 20,000	79 (23.7)	70.4
	RMB 20,001 to RMB 30,000	37 (11.1)	81.4
	≥RMB 30,001	18 (5.4)	86.8
	No budget	44 (13.2)	100
**Time of stay at the casino**			
	<6 hours	172 (51.5)	51.5
	6 to 12 hours	96 (28.7)	80.2
	1 day to 2 days	48 (14.4)	94.6
	>2 days	18 (5.4)	100

^a^RMB $1=US $ 0.14.

Regarding the duration spent in casinos, the following patterns were observed among the 334 respondents: 172 (51.5%) individuals spent <6 hours in casinos, 96 (28.7%) individuals dedicated 6 to 12 hours, 48 (14.4%) individuals engaged in gambling for 1 to 2 days, and 18 (5.4%) individuals extended their visits for >2 to 3 days in casinos. These findings elucidate the diverse levels of engagement and time commitment exhibited by the surveyed individuals during their casino experiences.

### Measurement Model

First, we tested the questionnaire for its reliability. The measurement of gambling behavior had a Cronbach α coefficient of 0.824 for gambling amount, 0.824 for gambling mood, 0.804 for social gambling, and 0.858 for gambling involvement, indicating a high level of reliability. Next, the scale was further validated through an exploratory factor analysis. The Kaiser-Meyer-Olkin measure of sampling adequacy was found to be 0.940, indicating that the scale was suitable for factor analysis. The Bartlett Test of Sphericity yielded a chi-square value of 2105.821 (*P*<.001), supporting the suitability of the scale for factor analysis. Principal component analysis was used for factor analysis using the maximum variance method. The dimensionality reduction analysis was performed using SPSS software (version 26.0; IBM Corp). The validity test demonstrated that each question factor had loadings ranging from 0.659 to 0.813, indicating a high level of conceptual validity for the scale. The total number of explanatory variables accounted for 72.65% of the variance, indicating that the measured indicators provided substantial explanatory power. Overall, the analysis revealed a high level of reliability of the measured indicators.

### Variance Analysis

We conducted binary logistic regression analysis based on unweighted data to examine group comparisons of gambling behaviors. Chi-square tests were used to explore potential independent variables associated with the changes in gambling behavior, which served as the dependent variable. The results of the logistic regression analysis are presented as odds ratios with 95% CIs. All statistical analyses were performed using SPSS software.

First, we analyzed the samples based on whether respondents of different genders participated in Macau casinos during the pandemic and engaged in online gambling. The descriptive statistics of the sample are presented in Table 2. A total of 64.4% (215/334) male individuals and 35.6% (119/334) female individuals responded to the survey. Among the 334 participants, a total of 175 (52.4%) respondents were involved in online gambling, of which 136 (77.7%) were male individuals and 39 (22.3%) were female individuals. During the pandemic, 78.4% (116/334) of male individuals and 21.6% (32/334) of female individuals were involved in online gambling. In addition, 27 (8.1%) of the 334 respondents, comprising 20 (74.1%) male individuals and 7 (25.9%) female individuals, did not visit Macau; 15 (4.5%) respondents, comprising 10 (66.7%) male individuals and 5 (33.3%) female individuals, traveled to Macau for gambling. During the pandemic, 171 (51.2%) of the 334 respondents, with 89 (52%) male individuals and 82 (48%) female individuals, did not travel to Macau. This shows that the impact of the pandemic on gambling is enormous.

**Table 2:** Survey respondents and the final study populations by gambling experienced group (N=334).

	GONLINE^a^											
	Yes				No							Total n (%)
	GINVOlVE^b^				GINVOlVE				Total			
	Yes (n=148), n (%)	No (n=27), n (%)	Total (n=175), n (%)	Yes (n=15), n (%)		No (n=144), n (%)	Total (n=159), n (%)	Yes (n=163), n (%)		No (n=171), n (%)		
Male individuals	116 (78.4)	20 (74.1)	136 (77.7)	10 (66.7)		69 (47.9)	79 (49.7)	126 (77.3)		89 (52)	215 (64.4)	
Female individuals	32 (21.6)	7 (25.9)	39 (22.3)	5 (33.3)		75 (52.1)	80 (50.3)	37 (22.7)		82 (48)	119 (35.6)	

^a^GONLINE: gambling online.

^b^GINVOlVE: gambling in casinos.

The basic statistics related to traveling to Macau and participating in online gambling are presented in Table 3. Gender differences were evident, with notable variations in gambling habits among individuals aged 21 years, frequency of gambling before the COVID-19 pandemic, monthly income, gambling budget (before the pandemic), gambling budget (during the pandemic), and the length of stay at casinos (*P*<.01). Furthermore, marital status exhibited significant differences in traveling to Macau for gambling (*P*<.05), whereas no notable differences were observed in online gambling. However, there were no significant differences in terms of education or age between those who traveled to Macau and those who engaged in online gambling.

An independent sample test was conducted to compare the gender of individuals who traveled to Macau for gambling (Table 4). The results revealed that there was a significant difference in the gambling amount for married individuals who traveled to Macau and participated in gambling during the pandemic (*P*<.05). However, no significant difference was observed in traveling to Macau for gambling during the pandemic. These findings suggest that the pandemic has had a significant impact on the gambling behavior of married gamblers. In addition, regardless of marital status, there were no significant differences in gambling amounts during the pandemic.

**Table 3:** Gambling behavior by demographic characteristics among all participants (N=334).

Item and comparison group			GINVOlVE^a^					*P* value			GONLINE^b^					*P* value
			Yes, n (%)		No, n (%)					Yes, n (%)			No, n (%)			
**Sex**								<.01								<.01
	Male individuals (n=215)	126 (58.6)		89 (41.4)					136 (63.3)			79 (36.7)				
	Female individuals (n=119)	37 (31.1)		82 (68.9)					39 (32.8)			80 (67.2)				
**Gambling before the age of 21 years**								<.01								<.01
	Yes (n=164)	135 (82.3)		29 (17.7)					139 (84.8)			25 (15.2)				
	No (n=170)	28 (16.5)		142 (83.5)					36 (21.2)			134 (78.8)				
**Education**								.90								.58
	High school or below (n=83)	42 (50.6)		41 (49.4)					47 (56.6)			36 (43.4)				
	Junior college (n=139)	66 (47.5)		73 (52.5)					73 (52.5)			66 (47.5)				
	Bachelor or above (n=112)	55 (49.1)		57 (50.9)					55 (49.1)			57 (50.9)				
**Habit**								<.01								<.01
	Smoking (n=91)	52 (57.1)		39 (42.9)					56 (61.5)			35 (38.5)				
	Drinking (n=102)	69 (67.6)		33 (32.4)					74 (72.5)			28 (27.5)				
	None of the above (n=141)	42 (29.8)		99 (70.2)					45 (31.9)			96 (68.1)				
**Marital status**								.01								.10
	Married (n=259)	136 (52.5)		123 (47.5)					142 (54.8)			117 (45.2)				
	Unmarried (n=75)	27 (36)		48 (64)					33 (44)			42 (56)				
**Age (years)**								.25								.24
	≤25 (n=46)	27 (58.7)		19 (41.3)					29 (63)			17 (37)				
	26 to 35 (n=192)	94 (49)		98 (51)					100 (52.1)			92 (47.9)				
	≥36 (n=96)	42 (43.8)		54 (56.3)					46 (47.9)			50 (52.1)				
**Gambling frequency (before pandemic)**								<.01								<.01
	Once a week (n=81)	55 (67.9)		26 (32.1)					61 (75.3)			20 (24.7)				
	Once a month (n=126)	80 (63.5)		46 (36.5)					86 (68.3)			40 (31.7)				
	Once a day (n=23)	16 (69.6)		7 (30.4)					13 (56.5)			10 (43.5)				
	Irregular (n=104)	12 (11.5)		92 (88.5)					15 (14.4)			89 (85.6)				
**Monthly income^c^**								<.01								.02
	≤RMB 10,000 (n=177)	69 (39)		108 (61)					79 (44.6)			98 (55.4)				
	RMB 10,001 to RMB 20,000 (n=95)	57 (60)		38 (40)					58 (61.1)			37 (38.9)				
	RMB 20,001 to RMB 30,000 (n=44)	28 (63.6)		16 (36.4)					29 (65.9)			15 (34.1)				
	≥RMB 30,001 (n=18)	9 (50)		9 (50)					9 (50)			9 (50)				
**Gambling budget (before the pandemic)**								<.01								<.01
	≤RMB 10,000 (n=202)	76 (37.6)		126 (62.4)					90 (44.6)			112 (55.4)				
	RMB 10,001 to RMB 20,000 (n=76)	47 (61.8)		29 (38.2)					45 (59.2)			31 (40.8)				
	RMB 20,001 to RMB 30,000 (n=45)	34 (75.6)		11 (24.4)					33 (73.3)			12 (26.7)				
	≥RMB 30,001 (n=11)	6 (54.5)		5 (45.5)					7 (63.6)			4 (36.4)				
**Gambling budget (during the pandemic)**								<.01								<.01
	≤RMB 10,000 (n=156)	76 (48.7)		80 (51.3)					86 (55.1)			70 (44.9)				
	RMB 10,001 to RMB 20,000 (n=79)	49 (62)		30 (38)					51 (64.6)			28 (35.4)				
	RMB 20,001 to RMB 30,000 (n=37)	20 (54.1)		17 (45.9)					20 (54.1)			17 (45.9)				
	≥RMB 30,001 (n=18)	16 (88.9)		2 (11.1)					15 (83.3)			3 (16.7)				
	No budget (n=44)	2 (4.5)		42 (95.5)					3 (6.8)			41 (93.2)				
**Time of stay at the casino**								.02								<.01
	<6 hours (n=172)	70 (40.7)		102 (59.3)					74 (43)			98 (57)				
	6 to 12 hours (n=96)	58 (60.4)		38 (39.6)					63 (65.6)			33 (34.4)				
	1 day to 2 days (n=48)	25 (52.1)		23 (47.9)					28 (58.3)			20 (41.7)				
	>2 days (n=18)	10 (55.6)		8 (44.4)					10 (55.6)			8 (44.4)				

^a^GINVOlVE: gambling in casinos.

^b^GONLINE: gambling online.

^c^RMB $1=US $1 0.14.

**Table 4:** Gambling behavior independence tests^a^.

Gambling in casinos				Levin test				Mean equivalence *t* test (2-tailed)				Mean squared error difference (SE)		95% CI
				*F* test (*df*)		*P* value		*t* test (*df*)		*P* value				
**No**														
	**AGB1** ^b^													
		Equal variance	4.923 (332)		.03		−2.717 (169)		.007		−0.396 (0.146)		−0.684 to −0.108	
**None**														
	Equal variance		—^c^		—		−3.089 (114.972)		.003		−0.396 (0.1284)		−0.651 to −0.142	

^a^Gambling behavior independence tests compared to unmarried.

^b^AGB1: gambling amount.

^c^Not available.

We conducted an ANOVA on the variables related to gambling in Macau (Table 5). The results indicated that individuals who traveled to Macau for gambling during the pandemic reported spending >2 days at casinos and gambling significantly higher amounts compared to those who stayed for 1 to 2 days. in addition, the social gambling behavior of those who stayed between 6 and 12 hours and those who stayed for >2 days was significantly higher than that of individuals who stayed for 1 day to 2 days.

The budget for each gambling session (before the pandemic) indicated notable differences among the different income groups. The group with a budget of ≥RMB 30,001 reported gambling significantly higher amounts compared to the group with a budget of RMB 20,001 to RMB 30,000 and the group with a budget of <10,000 or the group without a budget. There were significantly more gamblers aged <25 years and 26 to 35 years in Macau during the pandemic than those aged >36 years. Among gamblers who gambled more often than before the pandemic, the group that gambled once a day reported significantly higher rates of gambling than the other frequency groups.

Furthermore, an ANOVA was conducted on variables related to online gambling (Table 6). Regarding academic qualifications, the influence of gambling sentiment on gamblers with a college degree was considerably higher than that on gamblers with a high school education or below. In terms of the frequency of gambling before the pandemic, the group that gambled daily exhibited significantly higher rates compared to the other frequency groups. Among online gamblers, those aged 26 to 35 years accounted for a larger proportion of gambling compared to those aged >35 years; those aged <25 years and 26 to 35 years significantly engaged in more social gambling compared to those aged >35 years. Among non–online gamblers, those aged <25 years and those aged 26 to 35 years had a significantly larger presence than those in the 35-year age group.

Online gambling budgets vary during the COVID-19 pandemic. Nevertheless, the group with a gambling budget of >RMB 30,001 still maintained significantly higher gambling amounts compared to the group without a budget for online gambling. In terms of the duration spent at the casino participating in gambling, the group spending >2 days at the casino stayed more than twice as long as the group spending 1 day to 2 days.

**Table 5:** ANOVA multiple comparisons of the involved gambling behavior.

Gambling in casino, variable, and comparison group						Mean deviation		σ		*P* value		95% CI
**Time to stay at the casino**												
	**Yes**											
		**AGB1^a^**										
			**1 day to 2 days**									
				<6 hours	0.274		0.213		.20		−0.147 to 0.696	
				6 to 12 hours	0.308		0.219		.16		−0.125 to 0.741	
				>2 days	0.760^b^		0.343		.03		0.083 to 1.437	
		**AGB3^c^**										
			**1 day to 2 days**									
				<6 hours	0.390		0.199		.053		−0.004 to 0.783	
				6 to 12 hours	0.497^b^		0.205		.02		0.093 to 0.902	
				>2 days	0.880^b^		0.320		.007		0.248 to 1.513	
**Gambling budget (before the pandemic)**												
	**No**											
		**AGB1**										
			≤**RMB 10,000**^d^									
				RMB 10,001 to RMB 20,000	0.240		0.185		.20		−0.126 to 0.606	
				RMB 20,001 to RMB 30,000	−0.128		0.231		.58		−0.584 to 0.329	
				≥RMB 30,001	0.362^b^		0.620		.03		0.138 to 2.587	
				No budget	0.021		0.165		.90		−0.305 to 0.347	
			**RMB 20,001 to RMB 30,000**									
				≤RMB 10,000	0.128		0.231		.58		−0.329 to 0.584	
				RMB 10,001 to RMB 20,000	0.368		0.263		.16		−0.151 to 0.887	
				≥RMB 30,001	0.490^b^		0.647		.02		0.212 to 2.768	
				No budget	0.149		0.249		.55		−0.343 to 0.641	
			≥**RMB 30,001**									
				≤RMB 10,000	−0.362^b^		0.620		.03		−2.587 to −0.138	
				RMB 10,001 to RMB 20,000	−0.122		0.632		.08		−2.371 to 0.127	
				RMB 20,001 to RMB 30,000	−0.490^b^		0.647		.02		−2.768 to −0.212	
				No budget	−0.341^b^		0.627		.03		−2.579 to −0.104	
**Age (years)**												
	**Yes**											
		**AGB2^e^**										
			≥**36**									
				26 to 35	0.351^b^		0.170		.04		0.017 to 0.687	
				≤25	0.477^b^		0.212		.03		0.059 to 0.895	
**Gambling frequency (before the pandemic)**												
	**No**											
		**AGB1**										
			**Once a week**									
				Once a month	0.011		0.213		.96		−0.410 to 0.432	
				Once a day	0.739^b^		0.370		.047		0.009 to 1.471	
				Irregular	0.026		0.193		.90		−0.356 to 0.407	
			**Once a month**									
				Once a week	−0.011		0.213		.96		−0.432 to 0.410	
				Once a day	0.728^b^		0.353		.04		0.032 to 1.425	
				Irregular	0.014		0.157		.93		−0.296 to 0.325	
			**Once a day**									
				Once a week	−0.739^b^		0.370		.047		−1.471 to −0.009	
				Once a month	−0.728^b^		0.353		.04		−1.425 to −0.032	
				Irregular	−0.714^b^		0.341		.04		−1.387 to −0.041	

^a^AGB1: gambling amount.

^b^*P*<.05.

^c^AGB3: social gambling.

^d^RMB $1=US $1 0.14.

^e^AGB2: gambling mood.

**Table 6:** ANOVA multiple comparisons of the online gambling behavior least significant difference ANOVA analysis.

Gambling online, variable, and comparison group								Mean deviation		σ		*P* value		95% CI
**Education**														
	**Yes**													
		**AGB2^a^**												
				**High school or below**										
						Junior college	−0.340^b^		0.163		.04		−0.663 to −0.018	
						Bachelor’s degree or above	−0.268		0.173		.12		−0.610 to 0.074	
**Gambling frequency (before the pandemic)**														
	**Yes**													
		**AGB1^c^**												
				**Once a week**										
						Once a month	0.167		0.155		.28		−0.139 to 0.473	
						Once a day	0.663^b^		0.283		.02		0.105 to 1.222	
						Irregular	−0.042		0.267		.87		−0.569 to 0.485	
				**Irregular**										
						Once a week	0.042		0.267		.87		−0.485 to 0.569	
						Once a month	0.210		0.259		.42		−0.302 to 0.721	
						Once a day	0.706^b^		0.351		.046		0.013 to 1.399	
**Age (y)**														
	**Yes**													
		**AGB1**												
				≥**36**										
						≤25	0.356		0.220		.11		−0.078 to 0.790	
						26 to 35	0.350^b^		0.165		.04		0.024 to 0.676	
		**AGB2**												
				≥**36**										
						≤25	0.530^b^		0.205		.01		0.125 to 0.936	
						26 to 35	0.332^b^		0.154		.03		0.029 to 0.637	
		**AGB3** ^d^												
				≥**36**										
						≤25	0.411^b^		0.203		.045		0.010 to 0.813	
						26 to 35	0.393^b^		0.153		.01		0.092 to 0.695	
	**No**													
		**AGB1**												
				≤**25**										
						26 to 35	0.539^b^		0.223		.02		0.099 to 0.980	
						≥36	0.557^b^		0.237		.02		0.089 to 1.026	
**Gambling budget (before the pandemic)**														
	**Yes**													
		**AGB1**												
				**No budget**										
						≤RMB 10,000^e^	0.973		0.546		.08		−0.106 to 2.052	
						RMB 10,001 to RMB 20,000	0.922		0.553		.10		−0.170 to 2.013	
						RMB 20,001 to RMB 30,000	0.683		0.576		.24		−0.454 to 1.820	
						≥RMB 30,001	0.778^b^		0.588		.047		0.016 to 2.339	
**Time of stay at the casino**														
	**Yes**													
		**AGB3**												
				**>2 days**										
						<6 hours	−0.508		0.290		.08		−1.080 to 0.064	
						6 to 12 hours	−0.400		0.293		.17		−0.978 to 0.178	
						1 day to 2 days	−0.781^b^		0.317		.02		−1.407 to −0.155	

^a^AGB2: gambling mood.

^b^*P*<.05.

^c^AGB1: gambling amount.

^d^AGB3: social gambling.

^e^RMB $1=US $1 0.14.

## Discussion

### Principal Findings

Recognition is growing that gambling poses an increasing public health concern. Despite being beneficial for the economy and profitable for businesses, gambling causes personal and social harm to both individuals and communities. In particular, gambling presents a significant global public health challenge with the rise of online gambling [60]. The effects of gambling on public health are evident across various levels, including personal, interpersonal, and societal dimensions [61]. In this cross-sectional study, we investigated the changes in the gambling behavior of Mainland Chinese gamblers visiting Macau and participating in online gambling during the COVID-19 pandemic. In addition, we analyzed other factors that influenced gambling behavior during the pandemic, such as certain sociodemographic characteristics. We used data from a sample of 334 respondents to conduct a comparative analysis of gamblers’ gambling behaviors. The main findings of this study are presented subsequently.

First, the COVID-19 pandemic has had a significant and wide-ranging impact on gambling behavior. Among the 334 respondents in this study, a substantial proportion of 171 (51.2%) individuals reported not participating in gambling during the pandemic. This finding underscores the profound influence of the pandemic on individuals’ gambling behavior. Specifically, the willingness of gamblers to visit Macau for gambling tourism has been affected due to the various consequences of public health measures [56]. A notable observation from the study is that 89 male individuals and 171 female individuals discontinued their gambling activities, regardless of whether it involved traveling to Macau or participating in online gambling. This finding is consistent with previous studies conducted by Auer et al [22] and Linka et al [13], which highlight the fact that gamblers assess the risk associated with travel [14] and make decisions influenced by destination marketing strategies [15]. In addition, a prolonged lack of access to gambling facilities as a result of the pandemic has led to a gradual reduction in interest in gambling games or a modification of their gambling habits [16]. These changes can be interpreted as temporary alterations or even temporary abandonment of gambling habits as a direct consequence of their health consideration under public health measures.

Second, it is noteworthy that, among the 334 respondents, 175 (52.4%) individuals engaged in online gambling, with 136 (77.7%) being male individuals and 39 (22.3%) being female individuals. This indicates the proportion of participants who turned to online gambling, particularly during the pandemic. Notably, 20 (74%%) male individuals and 7 (26%) female individuals of the respondents refrained from gambling in Macau and instead opted for an alternative approach, which included online gambling. This finding aligns with the conclusions drawn by Sachdeva et al [16], indicating that individuals consider various factors that influence their ultimate gambling behavior in the face of changing circumstances, especially during a pandemic.

Third, the results revealed significant differences among Mainland Chinese gamblers who engaged in both travel to Macau and online gambling, with regard to gender, gambling habits, prepandemic frequency of play, monthly income, gambling budget (before and during the pandemic), and length of stay at the casino (*P*<.01). Notably, marital status demonstrated significant differences in terms of traveling to Macau for gambling (*P*<.05), whereas no significant differences in terms of marital status were observed in online gambling. However, we did not find any significant differences regarding the education and age of gamblers in this study. Despite the changes in participation methods and behaviors during the pandemic, this finding aligns with the conclusions drawn by Chan et al [50], suggesting that problem gambling may be prevalent among Chinese gamblers, regardless of whether they participate in gambling at land-based casinos or web-based platforms.

To examine the difference in gambling behavior among individuals who visited casinos, we found from this study that there was a significant difference in gambling amount among married individuals who traveled to Macau for gambling (*P*<.05). However, there was no significant difference in the gambling budget of married gamblers before and during the pandemic. Moreover, regardless of marital status, there was no significant difference in gambling expenditure during the pandemic. Among the gamblers who visited Macau for gambling during the pandemic, those who stayed longer at the casino and participated in gambling exhibited higher levels of gambling expenditure compared to those who stayed for 1 day to 2 days. This suggests that high-value gamblers tended to extend their stay at the casinos before the pandemic and had a tendency toward problem gambling [57]. Regarding casino gambling, respondents who stayed at the casinos for 6 to 12 hours or >2 days demonstrated a significantly greater level of involvement than those who stayed for 1 day to 2 days. Before the pandemic, individuals with gambling budgets of >RMB 30,001 were considerably higher than those with other budgets, indicating that high-value gamblers had higher budgets allocated to gambling before the pandemic. In terms of age demographics, gamblers aged <25 years and those aged 26 to 35 years were considerably more prevalent than those aged >36 years during the pandemic, indicating that young gamblers were more prevalent than gamblers in other stages before the pandemic. Among the gamblers who gambled regularly before the pandemic, those who gambled once a day exhibited a significantly higher frequency compared to those who gambled once a week, once a month, or irregularly. Furthermore, in the context of social gambling, it was observed that those aged <25 years and those aged 26 to 35 years had significantly higher frequency of gambling compared to those aged 35 years. This indicates a greater inclination of gamblers in these age groups to participate in gambling for social purposes [22]. Consequently, the pandemic has shaped their behaviors within the casino environment during this period of public health crisis.

When examining the difference in gambling behavior of online gamblers, the results reveal a significant difference in the number of gamblers based on educational attainment, particularly between college-educated individuals and those with a high school education or below. This discrepancy suggests that the gambling sentiment and mindset of gamblers have been influenced by the pandemic, leading to changes in their behavior. Regarding the frequency of gambling participation before the COVID-19 pandemic, the number of gamblers who gambled once a day was considerably higher than that in the other groups, suggesting that gamblers who gambled frequently also placed more bets. Regarding age, among the gamblers participating in online gambling, those aged 26 to 35 years exhibited significantly higher gambling expenditure compared to those aged 35 to 45 years; those aged <25 years and 26 to 35 years demonstrated significantly higher gambling mood compared to those aged 35 to 35 years; furthermore, those aged <25 years or 26 to 35 years displayed considerably high involvement in social gambling, indicating the increased participation of the younger generation in online gambling and social gambling activities compared to older age groups [22]. The budget allocation for each gambling session during the pandemic revealed that the group with a budget >RMB 30,001 was significantly larger than the group with other budget allocations, reflecting the importance of online gambling as a prevalent form of gambling in the aftermath of the pandemic. Regarding the duration of gambling participation, the group that engaged in gambling for >2 days was significantly larger than the group that participated for 1 day to 2 days. This finding suggests that online gambling serves as a platform that fulfills the socialization needs of gamblers over a prolonged period.

Gambling has gained widespread popularity among adolescents worldwide [23,62]. Excessive or problematic gambling can give rise to various public health implications, including financial consequences, addiction, and mental health issues. A public health approach is essential to address and mitigate the harm associated with gambling [63,64]. The harm caused by gambling can manifest in various ways, affecting both individuals and communities. Considering the economic significance of the gaming industry, it is advisable to adopt a comprehensive and coordinated approach that engages policy makers, gaming operators, and the broader community to effectively prevent problem gambling among both local citizens and tourists. Public health initiatives are recommended to involve the implementation of player protection measures, public awareness and education, provision of support services, self-regulation within the industry, and promotion of responsible gambling practices to create safer environments. Addressing gambling addiction issues at both personal and societal levels can lead to the minimization of negative impacts associated with gambling, the safeguarding of vulnerable populations, and the fostering of overall well-being in individuals and communities.

### Limitations

This study has several limitations that should be acknowledged. First, the data collected for this study were obtained from a survey conducted with gamblers on a social instant SMS text messaging platform (WeChat), which may introduce the possibility of selection bias and limit the generalizability of our findings. In addition, the relatively small sample size used in this study may restrict the generalizability of the results.

Second, the scale designed to measure gambling behavior in this study was simplified based on previous research. This simplification may have overlooked important dimensions of gambling behavior, potentially impacting the comprehensiveness of our findings. Furthermore, the study did not categorize the different types of gambling games, making it difficult to ascertain the specific preferences of gamblers, such as table games, slot machines, online gambling, and sports betting. Future research should consider using more comprehensive scales to capture the complexity of gambling behavior and incorporate the categorization of different types of gambling activities.

Third, while the cross-sectional design used in this study is useful for examining associations at a specific point in time, a time-series approach would provide a more accurate reflection of the dynamic changes in individuals’ gambling behavior. Future research should consider longitudinal designs to gain a better understanding of how gambling behaviors evolve and to capture the impact of various factors on these changes. Furthermore, it is important to recognize that the COVID-19 pandemic has significantly altered the macroenvironment, including work and income situations, which may have influenced changes in gambler behavior. Future research should include relevant control variables to account for these potential confounding factors and to obtain a more comprehensive understanding of the impact of the pandemic on gambling behavior.

### Conclusions

The primary conclusion of this paper is that a high proportion of the original Mainland Chinese gamblers opted not to visit Macau for gambling purposes after the outbreak of the COVID-19 pandemic. While the reasons for this shift may vary, the public health crisis has resulted in a decreased reliance on land-based gambling and a greater inclination toward online gambling. Consequently, there has been a reduction in the amount of money spent at gambling establishments and a decrease in the duration of casino visits. Casinos have traditionally served as highly popular social venues for gamblers. However, the antipandemic measures implemented by governments and casino operators have, to some extent, affected the social gambling behavior of individuals, leading to a shift away from traditional casinos and a greater emphasis on online gambling platforms.

Additionally, the COVID-19 pandemic has led to a notable increase in online gambling activities among Mainland Chinese gamblers. Particularly, the younger generations have been significantly affected by this observed phenomenon. Online gambling has gained popularity during the pandemic, with a substantial increase in both the amount and frequency of gambling observed. The restrictions imposed by the pandemic have had an impact on gamblers’ behavior, affecting their mood while participating in gambling activities. Notably, individuals with a middle level of education have experienced a significant impact on their gambling behavior. The findings of this study indicate that the pandemic has led to a decrease in land-based gambling, creating an environment that is conducive to controlling problem gambling; however, there appears to be a shift in gambling behavior from traditional casinos toward online gambling platforms as a direct consequence of the pandemic. The impact of the COVID-19 pandemic on gambling behavior underscores the significance of implementing targeted public health strategies and interventions to address the changing dynamics of gambling during public health emergencies.

## Abbreviations

SAR: Special Administrative Region

## Data Availability

The data supporting the findings of this study are available from the corresponding author upon reasonable request.
